# Treatment with Piribedil and Memantine Reduces Noise-Induced Loss of Inner Hair Cell Synaptic Ribbons

**DOI:** 10.1038/srep30821

**Published:** 2016-09-30

**Authors:** Richard A. Altschuler, Noel Wys, Diane Prieskorn, Cathy Martin, Susan DeRemer, Sanford Bledsoe, Josef M. Miller

**Affiliations:** 1 Kresge Hearing Research Institute, Department of Otolaryngology Head & Neck Surgery, University of Michigan, MI, USA; 2 Department of Cell & Developmental Biology, University of Michigan, MI, USA

## Abstract

Noise overstimulation can induce loss of synaptic ribbons associated with loss of Inner Hair Cell – Auditory Nerve synaptic connections. This study examined if systemic administration of Piribedil, a dopamine agonist that reduces the sound evoked auditory nerve compound action potential and/or Memantine, an NMDA receptor open channel blocker, would reduce noise-induced loss of Inner Hair Cell ribbons. Rats received systemic Memantine and/or Piribedil for 3 days before and 3 days after a 3 hour 4 kHz octave band noise at 117 dB (SPL). At 21 days following the noise there was a 26% and 38% loss of synaptic ribbons in regions 5.5 and 6.5 mm from apex, respectively, elevations in 4-, 8- and 20 kHz tonal ABR thresholds and reduced dynamic output at higher intensities of stimulation. Combined treatment with Piribedil and Memantine produced a significant reduction in the noise-induced loss of ribbons in both regions and changes in ABR sensitivity and dynamic responsiveness. Piribedil alone gave significant reduction in only the 5.5 mm region and Memantine alone did not reach significance in either region. Results identify treatments that could prevent the hearing loss and hearing disorders that result from noise-induced loss of Inner Hair Cell – Auditory Nerve synaptic connections.

Noise overstimulation results in over-release of glutamate from Inner Hair Cells (IHC) and excitotoxicity in post-synaptic peripheral processes of the Auditory Nerve (AN), with associated swelling, bursting and loss of effected afferent terminals (see refs 1, 2, 3 for reviews). Even a relatively mild noise overstimulation that does not result in outer hair cell (OHC) loss or a permanent shift in auditory brain stem response (ABR) thresholds, can result in a permanent loss of many IHC-AN synaptic connections and subsequently loss of spiral ganglion neurons (SGN)^4^. The mild noise overstimulation produced greatest loss in the sub-population of auditory nerve fibers with low spontaneous rates and low sensitivity, resulting in reduced dynamic range^5^. Recent studies suggest that loss of these IHC-AN synaptic connections can contribute to development of hyperacusis^6^ reduced Gap Detection^7^ as well as provide a trigger in the progression towards noise-induced chronic tinnitus^8^,^9^,^10^.

The present study assessed efficacy of systemic treatments with Piribedil and/or Memantine to reduce the noise-induced loss of IHC-AN synaptic connections. Piribedil is a dopamine agonist acting on lateral efferent synapses onto auditory nerve peripheral processes (15 for review). Previous studies have shown that intrascalar perfusion of Piribedil during noise exposure reduced compound action potential (CAP) threshold shifts and reduced excitotoxic damage to AN peripheral processes^11^,^12^. Garrett *et al*.^13^ also showed dopamine agonists could suppress CAP amplitudes. This may be accomplished by changing the “set-point” of auditory nerve peripheral processes that receive dopaminergic lateral efferent input^1^. Memantine is an NMDA receptor open channel blocker that has been shown to have anti-excitotoxic influence in other systems^14^,^15^,^16^,^17^,^18^,^19^. Systemic application of Piribedil or Memantine is currently in clinical use for other disorders (e.g. refs 20,21) and could be repurposed to prevent noise-induced excitotoxicity and loss of IHC-AN synaptic connections.

## Results

### Inner Hair Cell – Auditory Nerve Synaptic Ribbons

**Figure 1:**
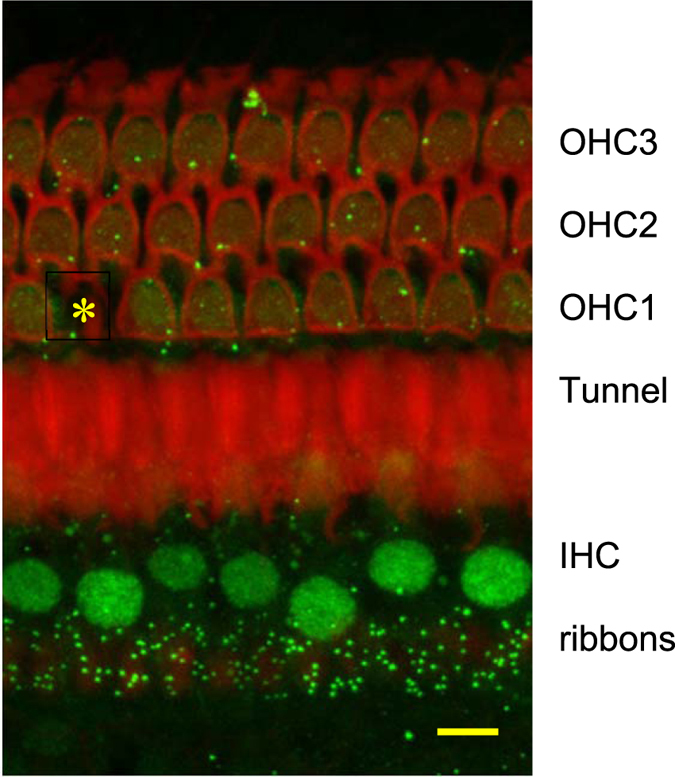
A representative laser scanning confocal digital image from a surface preparation of the cochlear spiral 5.5 mm from the apex, from a noise exposed rat without drug treatments. There is phalloidin labeling of f-actin (red), showing three rows of outer hair cells (OHC) and the tops of pillar cells of the tunnel. CTBP2 immunolabeling of ribbons (green) is seen under inner hair cells (IHC). There is also CTBP2 labeling of nuclei of IHC (green). There is scar (asterisk) replacing a missing outer hair cell (OHC) in the first row. Bar = 10 microns.

**Figure 2:**
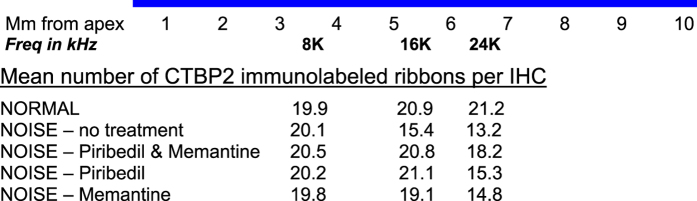
The mean number of CTBP2 immunolabeled ribbons per inner hair cell (IHC) for each of the three regions is shown for the five groups of rats assessed: NORMAL (no noise exposure, no drug treatments); NOISE – no treatment (4 kHz octave band noise exposure at 117 dB SPL for 3 hours and no drug treatments); NOISE – Piribedil and Memantine (noise exposure and treatment with both Piribedil and Memantine before and after the noise); NOISE – Piribedil (noise exposure and treatment with only Piribedil before and after the noise); NOISE –Memantine (noise exposure and treatment with only Memantine before and after the noise). The location of the three 0.2 mm regions along the ~10 mm average of the cochlear spiral is also shown as mm from the apex as well as the location of the three frequencies being tested by ABR (Viberg & Canlon 2004) (ABR threshold shifts are shown in Fig. 6).

**Figure 3:**
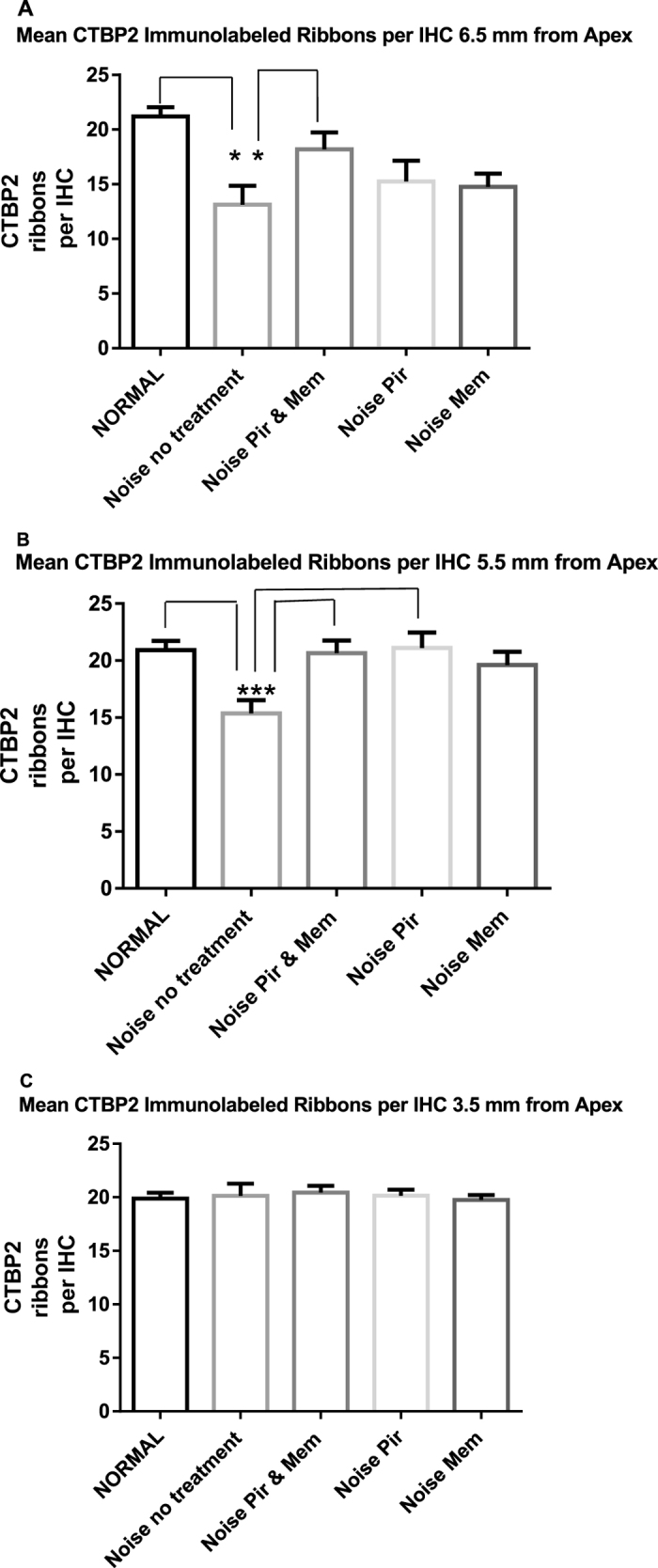
The mean number of CTBP2 immunolabeled ribbons per hair cell is graphed with standard error of the mean for five treatment conditions: NORMAL (no noise exposure, no drug treatments); Noise – no treatment (4 kHz octave band noise exposure at 117 dB SPL for 3 hours and no drug treatments); Noise Pir & Mm (noise exposure and treatment with both Piribedil and Memantine before and after the noise); Noise Pir (noise exposure and treatment with only Piribedil before and after the noise); Noise Mem (noise exposure and treatment with only Memantine before and after the noise) for ~0.2 mm regions assessed 6.5 mm from apex (2A), 5.5 mm from apex (2B) and 3.5 mm from apex (2C). (**A**) At 6.5 mm from apex significant differences are found between Normal and Noise exposed without treatment groups (p < 0.01) and between Noise exposed without treatment and Noise exposed with Piribedil and Memantine treatment groups (p < 0.05); (**B**) At 5.5 mm from apex significant differences are found between Normal and Noise exposed without treatment groups (p < 0.01), between Noise exposed without treatment and Noise exposed with Piribedil and Memantine treatment groups (p < 0.01) and between Noise exposed without treatment and Noise exposed with Piribedil treatment groups (p < 0.01); (**C**) At 3.5 mm from apex no significant differences are found. Asterisks show significance.

Figure 1 shows a representative image of CTBP2 immunolabeling of IHC synaptic ribbons in a noise exposed animal without drug treatment. In non-treated animals, the three hour 117 dB Octave Band Noise centered at 4 kHz produced a significant (*p* < 0.01) loss of IHC synaptic ribbons at 21 days following the exposure in two of the three regions of the cochlear spiral assessed, at 5.5 mm from the apex (26% decrease) and 6.5 mm from the apex (38% decrease) but not at 3.5 mm from the apex (Figs 2 and 3A–C). Combined treatment with Piribedil and Memantine produced a significant (*p* < 0.05) reduction in the noise-induced loss of ribbons in the 5.5 and 6.5 mm regions. Treatment with just Piribedil gave a significant reduction in the loss in the 5.5 mm region (*p* < 0.05) and a trend towards reduction in the 6.5 mm region. Treatment with just Memantine resulted in a non-significant trend towards reduced loss of ribbons in the 5.5 and 6.5 mm regions.

#### 6.5 mm from apex

In the region 6.5 mm from the apex the normal mean number of synaptic ribbons per IHC base was 21.2 +/− 0.8. This was significantly decreased to 13.2 +/− 1.7 (*p* < 0.01) in the noise exposed animals without drug treatments. In the group of rats receiving treatment with Piribedil alone, following noise, there was a mean number of synaptic ribbons per IHC of 15.3 +/− 1.9 while for the group receiving Memantine alone the mean density was 14.8 +/− 1.2 synaptic ribbons per IHC. The increase in ribbon density over the untreated noise exposed group was not significant for either of these treatment conditions. In the group of animals receiving a combined Piribedil and Memantine treatment the mean number of synaptic ribbons per IHC was 18.2 +/− 1.5. This was significantly greater than the mean in untreated noise exposed animals (*p* < 0.05) and was not significantly different than the mean number in non-exposed, normal rats (Fig. 3A).

#### 5.5 mm from apex. 

In the region 5.5 mm from the apex the normal mean number of synaptic ribbons per IHC was 20.9 +/− 0.7, this was significantly decreased to 15.4 +/− 1.2 (*p* < 0.01) in the group of noise exposed animals without drug treatments. The group of rats receiving treatment with Piribedil alone had a mean number of synaptic ribbons per IHC of 21.1 +/− 1.3, a significant increase (*p* < 0.01) over the number found in the noise exposed without drug treatment group and not significantly different from normal. The group receiving Memantine alone had a mean of 19.1 +/− 1.5 synaptic ribbons per IHC, close to but not reaching a significant difference (*p* = 0.07) from the noise exposed without drug treatment group. In the group of animals receiving a combined Piribedil and Memantine treatment the mean number of synaptic ribbons per IHC was 20.8 +/− 1.0. This was significantly greater than the mean in untreated noise exposed animals (*p* < 0.01) and was not significantly different than the mean number in normal rats (Fig. 3B).

#### 3.5 mm from apex. 

In the region 3.5 mm from the apex there was no significant noise-induced decrease in the mean number of synaptic ribbons, with a mean of 19.9 +/− 0.5 in normal animals and a mean of 20.1+/ = 0.9 in noise exposed animals without drug treatments. There was no significant treatment effect, with a mean of 20.1 +/− 0.6 in noise exposed animals treated with Piribedil, a mean of 19.8 +/− 0.5 in noise exposed animals treated with Memantine and a mean of 20.4 +/− 0.6 in noise exposed animals treated with Piribedil and Memantine (Fig. 3C).

### Hair Cells

**Figure 4:**
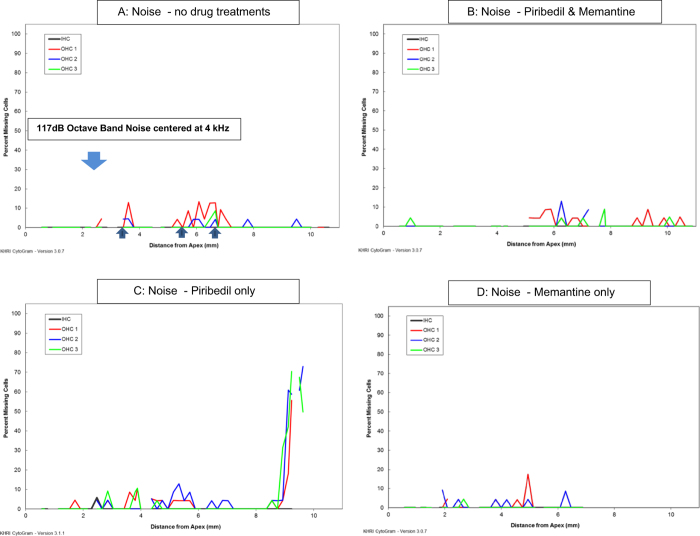
Representative cytocochleograms from the four noise treatment conditions, mapping loss of inner and outer hair cells along the length of the cochlear spiral in (**A**). Cytocochleogram from a rat in the noise exposure without drug treatment group. There is a small loss of outer hair cells (OHC) in the 3–4 and 5–7 mm from apex regions, greatest in the first row (red line). There is no loss of inner hair cells (IHC). The large blue arrow pointing down shows the approximate location of the 117 dB Octave Band Noise exposure centered at 4 kHz. The smaller and darker blue arrows pointing up show the location of regions where the number of CTBP2 immunolabeled ribbons per inner hair cell were assessed. (**B**) Cytocochleogram from a rat in the noise exposure group with Memantine and Piribedil drug treatment. There is minimal loss of OHC and no loss of IHC. (**C**) Cytocochleogram from a rat in the noise exposure group with Piribedil drug treatment. There is minimal loss of OHC except for a region of large loss in the most basal portion of the cochlear spiral. There is no loss of IHC. (**D**) Cytocochleogram from a rat in the noise exposure group with Memantine drug treatment. There is a small loss of OHC in region 5 mm from apex, there is no loss of IHC.

The three hours of 117 dB Octave Band Noise centered at 4 kHz produced only a small loss of OHCs in non-treated animals, with a mean loss of 2.2% of OHCs across turns and minimal loss of IHCs (less than 0.2%) occurring in only a few animals. Figure 4A shows a representative cytocochleogram mapping hair cell loss in a noise exposed rat that received no drug treatments, Fig. 4B–D show representative cytocochleograms from noise exposed animals with the different drug treatments. Typical of noise, the location of loss had considerable variability across exposed animals. There was no drug-treatment effect on the total mean hair cell losses observed in the noise exposed animals receiving any of the three drug treatments, little or no inner hair cell loss was observed in any group and the mean loss of OHCs varied between 1.4 and 3% across all groups.

### ABR Thresholds

**Figure 5:**
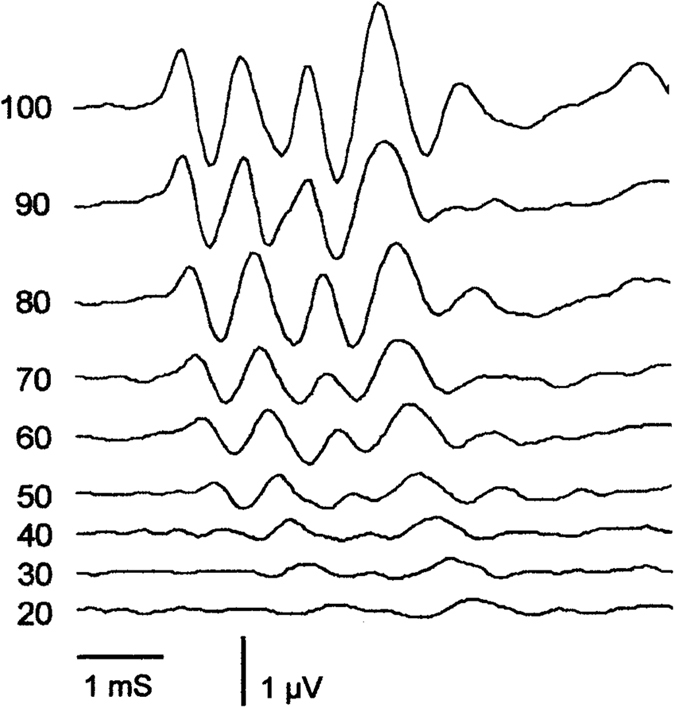
Auditory Brain Stem Response (ABR) waveforms in response to tonal stimuli at 20 kHz and intensities in 10 dB steps from threshold to near saturation.

**Figure 6:**
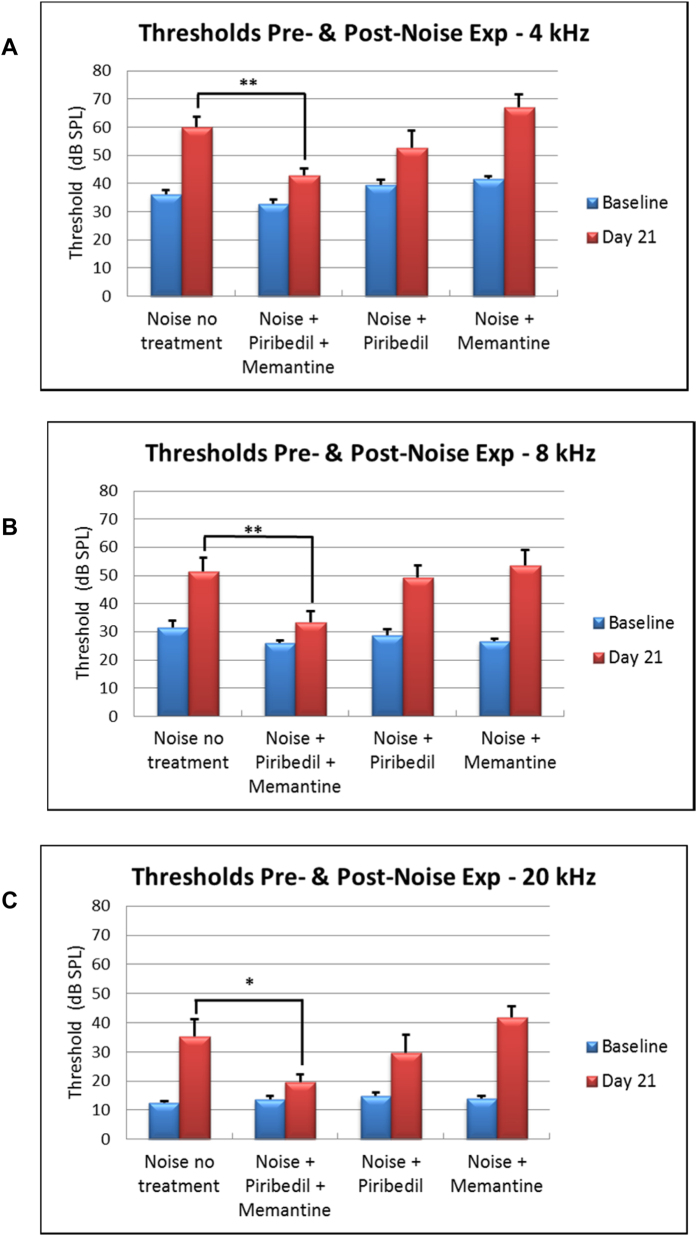
Threshold sensitivity in dB SPL of tonal ABRs to 4- (Fig. 4A), 8- (Fig. 4B), and 20 kHz (Fig. 4C) stimulation at Baseline (pre-noise exposure) and Day 21 following noise exposure for four groups of animals: Untreated Controls, treatment with Piribedil plus Memantine, treatment with Piribedil alone, and treament with Memantine alone. A significant (*p* < 0.01) increase in threshold at Day 21 compared to Baseline was seen following noise exposure in the Untreated Controls at all frequencies tested. There was no significant difference at in the threshold shift at 21 days following noise in the group treated with Piribedil plus Memantine or the group treated with Piribedil only compared to untreated controls, however there was a significant difference in the threshold shift in the Piribedil plus Memantine group compared to the Untreated Controls.

Figure 5 shows normal ABR waveforms to tonal stimuli at 20 kHz and intensities in 10 dB steps from threshold to near saturation. Figure 6 shows the baseline and day 21 post-noise exposure threshold measurements at 4-, 8- and 20 kHz for all groups. Baseline thresholds were similar across all groups, albeit with some elevation in the Piribedil-alone and Memantine-alone treated groups at the lowest frequency tested (4 kHz, Fig. 6). In the no-drug-treatment group the ABR thresholds at 21 days after the noise were significantly elevated over the baseline value, by 23 dB (*p* < 0.01), 20 dB (*p* < 0.01) and 23 dB (*p* < 0.01) at 4-, 8-, and 20 kHz respectively. The mean post-noise exposure thresholds in those groups treated with Piribedil-alone or Memantine-alone were significantly (p > 0.05) elevated over baseline at all frequencies tested, except for 4 kHz Piribedil-alone; and for these two single drug treatment groups the post-exposure thresholds were not significantly decreased from that observed in the group receiving noise but no drug treatment at any frequency tested. The group receiving combined Piribedil and Memantine treatment showed a significant (*p* < 0.05) decrease in noise-induced threshold shift compared to the group without drug treatment at 4 kHz (10 dB vs 24 dB), 8 kHz (7 dB vs 20 dB), and 20 kHz (6 dB vs 23 dB).

### ABR I/O Functions

**Figure 7:**
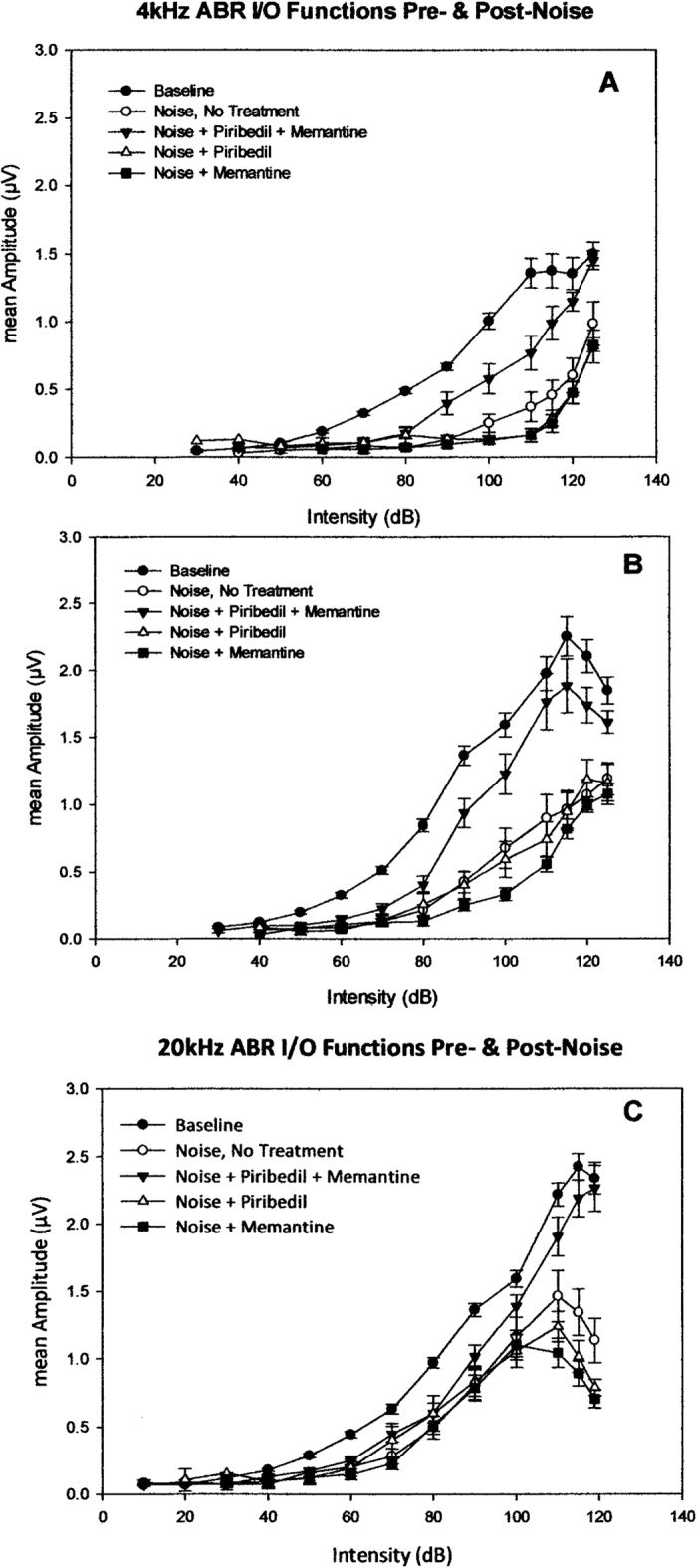
Mean ABR input/output functions to tonal stimuli at 4-, 8- & 20 kHz (**A–C**, respectively) at baseline (pre-noise exposure) and Day 21 following noise exposure for four groups of animals: Untreated Controls, treated with Piribedil plus Memantine, treated with Piribedil alone, and treated with Memantine alone. The baseline I/O function is mean of all animals prior to noise exposure. The greatest reduction in the slopes and dynamic range of responsiveness is seen in the group treated with Piribedil alone and Memantine alone. Combined Piribedil plus Memantine treatment maintained the responsiveness, most readily seen at 20- and 8 kHz frequencies, the two most basal sites, consistent with the maintenance of preservation of ribbon at the two most basal sites.

**Figure 8:**
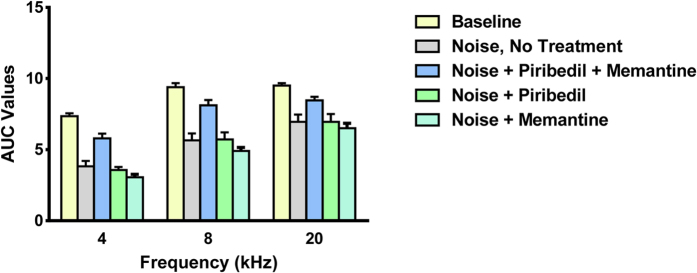
Analysis of the significance of the differences in the ‘area under the curve’ (AUC) between baseline and Day 21 for each of the groups showed a significant difference for the two single drug treatment groups (*p* < 0.05) and no difference for the group treated with Piribedil plus Memantine at 8 and 20 kHz.

Figure 7A–C show the mean ABR I/O functions obtained at 4-, 8- and 20 kHz, respectively, prior to noise exposure (baseline), including all animals in study, and at day 21 following noise exposure of each treatment group, including: no-treatment, treatment with Piribedil + Memantine, Piribedil-alone, and Memantine-alone. In all cases, following noise the functions are shifted to the right (higher intensity required for an equivalent response), with a reduction in the maximum amplitude of response observed. To quantify these changes, we assessed the mean area under the curve (AUC) for each function, to best reflect the change in dynamic range of responsiveness, and performed an ANOVA, on the mean AUC values. Figure 8 is a graph of the AUC values and SEMs. The reduction in responsiveness following noise exposure, compared to baseline, was greatest and significant (*p* ≤ 0.01) in the untreated animals, and animals treated with Piribedil-alone and Memantine-alone at all frequencies. No significant noise-induced-reduction in responsiveness was observed in animals treated with Pirbedil + Memantine with the exception of 4 kHz (*p* = 0.001). There was a significant difference (*p* ≤ 0.01) between Piribedil + Memantine treatment vs. Memantine alone at all frequencies and Piribedil alone at 4 & 8 kHz.

## Discussion

A result of the current study, finding systemic Piribedil can reduce the noise-induced loss of IHC ribbons, is consistent with an earlier study using intrascalar application of Piribedil during a noise exposure that found a reduced bursting of auditory nerve peripheral processes^2^,^11^,^12^. The present study showed Piribedil treatment produced a significant reduction in ribbon loss only in the region 5.5 mm from apex (where there was a 26% loss of connections in non-treated rats), while in the region 6.5 mm from apex (where there was a 38% loss in the non-treated rats) the reduction was not sufficient to reach significance. Memantine treatment did not produce sufficient reduction of loss to reach significance in any region, coming close (*p* = 0.07) in the region 5.5 from apex and not in the 6.5 mm region. The greater loss in the 6.5 mm region suggests this region is more stressed by the noise exposure or that it is more sensitive to stress and this could be responsible for making either drug treatment alone insufficient. The combination of Piribedil and Memantine treatments significantly reduced the noise-induced loss of ribbons in the 6.5 mm region. This could reflect a synergistic interaction of two different mechanisms affected by each agent; however it is possible that either Piribedil or Memantine alone could be sufficient at an appropriate dose. It would be useful to generate dose-response measures for each agent for different regions of the cochlear spiral in future studies. It would also be useful to compare noise exposures with different stress spectrums.

The significant reduction in noise-induced loss of ribbons in the 6.5 mm region with Piribedil and Memantine treatment was associated with a significant reduction in the noise-induced loss of suprathreshold amplitudes (Fig. 7). This is consistent with previous studies showing that loss of IHC-AN synaptic connections results in changes in growth functions and suprathreshold I/O responses (e.g. ref. 5). This suggests that combined Pirbedil and Memantine treatment produced sparing of the noise-induced loss of low spontaneous rate fiber connections, reducing loss of dynamic range. It is interesting that the Pirbedil-Memantine combination treatment also produced a significant reduction in threshold shift. Given that there was no significant reduction in hair cell loss, the reduction in threshold shift could reflect some sparing of medium and high spontaneous rate fibers whose noise-induced loss would increase thresholds. It could also reflect dysfunction in remaining outer hair cells. Distortion product evoked otoacoustic emissions (DPOAE) were available in six animals that had minimal OHC loss (under 5%) and ABR threshold shifts over 30 dB. Three of these animals had minimal (0–10 dB) DPOAE shifts consistent with an effect on medium and low threshold fibers while the other three had 15–40 dB DPOAE shifts consistent with some remaining OHCs having had loss of function. It would be interesting in the future to specifically assess high- versus medium- and low-spontaneous rate fibers, if specific markers become available. It would also be interesting in future studies to determine if reducing the loss of connections, as accomplished in the current study, influences the development of hyperacusis or tinnitus that has been associated with loss of ribbons and Inner Hair Cell – Auditory Nerve synaptic connection in recent studies^6^,^9^,^10^.

The noise exposure in the current study only produced significant loss of synaptic ribbons in the two more basal regions of the cochlear spiral assessed. There was no significant loss of IHC ribbons 21 days after the noise in the more apical region of the cochlear spiral where the noise exposure was centered. This might reflect a difference in the stress and/or sensitivity to stress in different regions of the cochlear spiral. It is also interesting to consider data showing that higher than normal levels of NT-3 in the cochleae of transgenic mice prior to a mild noise exposure resulted in a significant regeneration and recovery of lost IHC-AN synaptic connections^22^. NT-3 has been shown to have an apex-base concentration gradient in the cochlea with higher levels more apically^23^,^24^,^25^. Thus it is possible that the endogenous levels of NT-3 in the more apical regions are sufficient to induce some recovery of lost connections. It might, therefore, be of value to examine noise-induced changes in IHC-AN synaptic connections at earlier times than the 21 days after noise assessed in the current study and determine if there is possible regeneration of connections. If so, it would be interesting to examine potential protection from the loss at earlier times after noise exposure.

The significant noise-induced reduction in the dynamic range of ABR response (Fig. 7A–C) in both untreated animals and those treated with Piribedil-alone and Memantine-alone is consistent with previous studies showing that loss of IHC-AN synaptic connections results in changes in growth functions and lower supra-threshold responses^5^. The lack of a significant reduction in this responsiveness (at higher frequencies) in the group treated with Piribedil + Memantine is consistent with protection from noise-induced loss of IHC-AN synaptic connections.

The I/O function assessments were based on measures of the ABR Wave I, with the area under this wave well correlated with the number of active auditory nerve fibers (see ref. 26 for review). While reduction in the amplitude of response to high intensity stimulation may reflect selective damage to low-spontaneous-rate-high-threshold synapses and fibers^5^; at these high intensities there is also significant activation of low- and medium-threshold fibers from more basal regions of the cochlea. These fibers contribute to the amplitude of the ABR wave and their contribution to high intensity responses in the normal animal and these noise-induced changes in responsiveness following noise needs further elucidation.

## Methods

### Subjects

Male Sprague Dawley rats were obtained from Charles River. Only healthy subjects with normal hearing as assessed by auditory brainstem response (ABR) were included in the study. Body weights ranged from 306–385 grams at study onset. This study was approved by the University Committee on Use and Care of Animals at the University of Michigan. The animal care and use program conforms to the standards of “The Guide for the Care and Use of Laboratory Animals”, revised 2011.

### Experimental Groups

Rats were randomly assigned to four experimental groups: 1) noise exposure with saline injections (n = 11); 2) noise exposure with both piribedil and memantine injections (n = 9); 3) noise exposure with piribedil injections (n = 6); or 4) noise exposure with memantine injections (n = 9). The number of subjects across groups varied as some animals had to be eliminated due to methodological reasons during histological assessment. CTBP2 density in these three groups was compared to our normal CTBP2 database including sham noise exposed animals (n = 15). Saline (SC), Piribedil 10 mg/kg (SC) and/or Memantine 3 mg/kg (SC) were administered for a total of 7 consecutive days; beginning 3 days before and 1 hour prior to noise exposure. Dosages were based on Chen *et al*.^27^, Kutzing *et al*.^16^ and Wroge *et al*.^19^. Saline injections (SC) were administered using an equivalent volume as was used for the Piribedil and/or Memantine groups.

### Noise Exposures

The experimental groups of subjects were exposed to a 4 kHz octave band noise at an equivalent continuous sound pressure level of 117 dB (SPL) in a ventilated sound exposure chamber for 3 hours. The noise exposure was selected to produce a mild loss of OHCs in the mid-range of the rat cochlea. The sound chamber was fitted with speakers (JBL: Model 2450H) driven by a power amplifier (Parasound: HCA-750). The amplifier signal input source was an audio CD player (Marantz: PMD320). The audio CD was created with sound editing software (Adobe: Audition 1.5). Sound levels were calibrated and the sound spectrum verified with a spectrum analyzer (Stanford Research Systems: SR760) and microphone (Bruel and Kjaer: Type 4136) at multiple locations within the sound chamber to ensure uniformity of the stimulus. The stimulus intensity varied by a maximum of 3 dB across measured sites within the exposure chamber. The sound level was verified before and after the noise exposure using a sound level meter (Quest Instruments: model 2200). The sound level meter microphone was positioned above the cages and calibrated to record at the level of the animal’s head during the noise exposure.

### Auditory Brain Stem Response

Subjects were acclimated for a minimum of 72 hours prior to baseline ABR. Animals were anesthetized with xylazine 10 mg/kg and ketamine 40 mg/kg IM and placed within an electrically and acoustically shielded chamber (CA-Tegner AB, Sweden) on a water-circulating heating pad to maintain body temperature. Tucker Davis Technologies (TDT) System III hardware and SigGen/BioSig software (TDT, Alachua, FL) were used to present the stimulus and record responses. Neural activity was collected via subcutaneously inserted needle electrodes placed at the vertex of the skull (active) and ventral to each pinna. Input/Output (I/O) functions were measured in response to 15 mS tone bursts via a transducer inserted at the entrance to the ear canal; starting at an intensity of 80 dB, decreasing at 10 dB intervals until threshold was determined, then intensity was increased from 90 dB in 10 dB steps until 110 dB, followed by 5 dB steps to a maximum intensity of 120 dB at 20 kHz and 125 dB at 8 and then 4 kHz. A consistent 1024 responses were averaged for each of the stimulus intensities. Additional ABR collection details may be found in Le Prell *et al*.^26^,^27^. ABR measures with I/O functions were collected prior to inclusion in the study and again at 21 days post-noise exposure, prior to animal termination.

### Area Under the Curve

To determine the AUC, the measured amplitude (wave I from the input output functions) at each intensity level was added to the amplitude at the next lowest level and divided by 2. This product was multiplied by the difference between intensity levels (either 5 or 10 dB). These results were summed across intensities for a given subject, and the square root of each number was determined. The square roots were averaged across each group and significance was determined using ANOVA with Dunnett’s multiple comparison test.

### Cochlear Histolology and Immunostaining

Rats were heavily anesthetized with sodium pentobarbital and then received vascular perfusion through the heart with phosphate buffer followed by 4% Paraformaldehyde fixative in phosphate buffer. Cochleae were removed and received gentle intrascalar infusion of the same fixative through the round window and an apical fenestra, followed by immersion in fixative for 12–16 hours at 4 °C. Cochleae were then rinsed for 30 minutes in cold phosphate buffered saline (PBS) followed by partial decalcification in 5% EDTA in phosphate buffer for 2–3 days at room temperature on a rotator. The otic capsule was then removed and cochleae placed in 3% normal goat serum in PBS plus 0.3% Triton-X 100 as a blocking step. Tissues received primary incubation with mouse anti-CTBP2 antibody (BD Transduction Laboratories) diluted 1:200 in PBS plus 0.1% tritonX-100 for 16–20 h at 4 °C, followed by three ten minute rinses in PBS. Tissues were then co-labelled with both goat anti-mouse immunoglobulin with an Alexafluor 488 fluorescent label (Molecular Probes/Invitrogen) diluted 1:1000 in PBS – triton X and with phalloidin (Molecular Probes/Invitrogen) with an Alexafluor 568 fluorescent label, diluted 1:100 for 2 h at room temperature, followed by three rinses in PBS, in the dark. The cochleae were then microdissected into three segments, apex, base and hook, and each segment was mounted separately as a surface preparation on a glass slide with Prolong Diamond (Life Technologies) and covered with a coverslip. Slides were stored at 4 °C until examination.

### Assessment of Inner Hair Cell – Auditory Nerve Synaptic Ribbons

CTBP2 immunostaining of synaptic ribbons (Fig. 1) was used as a marker for IHC-AN synapses as in Kujawa and Liberman^4^, Singer *et al*.^10^ and Altschuler *et al*.^7^. Recent studies suggest that up to 5% of CTBP2 immuno-labeled ribbons can be “orphans” and not apposed by peripheral processes with glutamate receptors and so the use of CTBP2 immunostaining of ribbons as a marker for IHC-AN synaptic connections has the potential for an over-count of up to 5%^28^.

Three regions of interest were selected along the cochlear spiral at 3.5, 5.5 and 6.5 mm from the apex for quantitative assessment, close to the 8 and 20 kHz frequency regions^29^ respectively of our ABR assessments. An Olympus FluoView 500 Laser Scanning Confocal Microscope with a 63x objective was used to acquire a z-series with 1 μm slices at 0.25 μm intervals for each region of interest. The length of cochlear spiral in each region of interest was approximately 0.2 mm, varying because of differences in the curvature of the cochlear spiral at different positions. The digital images were imported into a Metamorph Image Analysis workstation for quantitative analysis which was blinded to treatment condition. The number of CTBP2 immuno-labeled puncta meeting size and shape criteria and with intensity of labeling at least five times over background was determined for each IHC base in the region of interest and then the mean number of puncta per IHC base in the region of interest was determined.

### Assessment of Hair Cell Loss

Phalloidin labeling of hair cells or of the scars replacing missing hair cells (Fig. 1) was used to identify presence or absence of IHCs and OHCs. Hair cells were counted under epifluorescence optics on a Leica fluorescent microscope using a 50x objective and a 0.19 mm reticule in the microscope eyepiece. The number of OHCs in each row and the number of IHCs that were present or absent for each 0.19 mm reticule length was entered into a cytocochleogram program (developed in house e.g. refs 30,31) starting at the apex and moving basally until the entire length of the cochlear spiral (9.7 mm average) had been assessed. The program compares hair cell numbers to a normal data base. The program can generate a graph of hair cell loss by position along the cochlear spiral for each cochlea (cytocochleogram) and also provide the analysis in absolute numbers or as the total percent of hair cells lost in each animal assessed.

## Statistics

Significance was tested by ANOVA (Kruskal-Wallis test for nonparametric results) followed by two-tailed unpaired t-test with Bonferonni correction for multiple comparisons, using Graphpad Prism.

## Additional Information

**How to cite this article**: Altschuler, R. A. *et al*. Treatment with Piribedil and Memantine Reduces Noise-Induced Loss of Inner Hair Cell Synaptic Ribbons. *Sci. Rep*. **6**, 30821; doi: 10.1038/srep30821 (2016).
